# Survey of Pet Owner Attitudes on Diet Choices and Feeding Practices for Their Pets in Portugal

**DOI:** 10.3390/ani12202775

**Published:** 2022-10-14

**Authors:** Joana C. Prata

**Affiliations:** 1 TOXRUN—Toxicology Research Unit, CESPU, University Institute of Health Sciences (IUCS), 3810-193 Gandra, Portugal; joanacorreiaprata@gmail.com; 2 O Meu Animal, 4515-463 Porto, Portugal

**Keywords:** companion animals, pet food preferences, pet food choices, feeding habits, alternative diets, natural pet food, organic pet food

## Abstract

**Simple Summary:**

New challenges for veterinarians and the pet food industry arise from emerging trends in pet foods, motivated by changes in pet owner’s beliefs and choices. Despite external influences, pet owners are the ones who ultimately decide on what and how to feed their pets. Therefore, a better understanding of pet food trends can be achieved by investigating motivations and husbandry practices related to feeding and diet. The objective of this study was to identify trends in pet food in Portugal using a preliminary survey. While most pets are fed commercial diets, there is a trend for an increasing interest in alternative diets, especially for organic and natural pet diets. This preference change is likely motivated by a greater interest in ingredients used in pet food, especially supporting a greater use of meat and lower use of carbohydrate sources. Therefore, alternative diets should be the focus of more recognition in research, pet food industry, and veterinarian practice.

**Abstract:**

Feeding practices and perceptions of pet owners determine consumer decisions on pet foods and influence the health of companion animals. The objective of the survey was to study emerging trends on pet food diets in Portugal by conducting a preliminary survey. A survey of 74 pet owners revealed that most fed pets with commercial diets (67.6%) bought in supermarkets (40.3%), spending a monthly average of EUR 30, following healthy practices (e.g., ≥30 min of exercise), which translated to an average self-reported pet body condition score of 3. Information about pet foods mainly originates from the animal’s veterinarian (64.9%), followed by the internet (16.2%). A trend for a growing interest in alternative diets (e.g., natural and organic) was identified, being already the second most consumed diet type (19.0%), perceived as being of a higher quality, and as a topic of interest (38.6%). This interest likely originates from a higher weighting of the ingredient list (31.5%) in consumer choices and beliefs that commercial diets should consist of a higher proportion of meat (29.3%) and less carbohydrates (38.7%). Therefore, more research is needed on the nutritional adequacy of alternative diets and uncommon ingredients, the pet food industry will have to adapt to changing consumer behaviors, and veterinarians should be available to discuss and oversee novel dietary practices in companion animals.

## 1. Introduction

Over half of the Portuguese homes are inhabited by companion animals [1], comprising a population of 2 million dogs and 1.4 million cats [2]. A previous survey conducted in Portugal identified diet as a major topic of interest for pet owners (39.6%), along with welfare (47.7%) and health assessment (45.0%) [3]. In the same study, 10.1% of respondents reported using alternative or homemade diets, as opposed to commonly used over-the-counter commercial pet foods. A previous study conducted in Italy revealed that pet foods may be following human nutrition trends, with consumers seeking a more “natural” diet, also reflecting a closer relationship and possible anthropomorphism of pets [4]. While human nutrition trends may complicate pet food choices, these often translate into a growing concern of nutrition’s influence on health [5]. This trend is accompanied by a growing mistrust of commercial pet foods, either because of their composition, processing, or perceived corrupt industry [6]. The concept of a “natural” diet often includes expectations over the replication of ancestral diets of cats and dogs [7]. Moreover, advocates of alternative diets (e.g., organic, vegetarian, grain free, and raw meat) often make unverified claims over their health benefits. For instance, proponents claim raw meat diets are nutritionally better and more palatable and maintain cleaner teeth, all anecdotal compared to the recognized dangers related to microbial contamination [8]. Similarly, grain free diets have been linked to cardiomyopathy cases in dogs [9]. Alternative diets may also raise sustainability concerns by competing for human ingredients instead of using by-products and by including a higher percentage of animal protein [10]. These alternative diets include vegetarian or vegan diets, which are plant-based [11]; raw meat diets, primarily based on uncooked animal ingredients [12]; homemade diets, which are formulated and cooked at home [13]; grain free diets, which exclude the use of grains; and natural and organic diets, comprising formulations which control for the type and origin of ingredients (e.g., excluding transgenics organism) and avoid the use of synthetic preservatives [6]. Other aspects of the dietary management of companion animals must be also taken into consideration due to their potential influence on health [6]. For instance, only 25.5% of Italian pet owners obtain information on pet food choices from their veterinarians [4]. In the USA, cat owners report mostly feeding dry food and only 30% have used food puzzles, which are important environmental enrichment tools [14]. Understanding feeding practices and pet owners’ motivations and perceptions are important not only to understand consumer decision but also for veterinarians to provide adequate advice and follow up [5]. However, feeding practices and opinions of Portuguese pet owners have not yet been explored. Therefore, the objective of this work was to study emerging trends on companion animal diets in Portugal by conducting a preliminary investigation on feeding practices and pet owners’ attitudes.

## 2. Materials and Methods

A survey consisting of 44 questions (Appendix A), with an estimated time of completion of 25 min, was developed to obtain information on regions, pets, diet choices, and opinions on nutrition or diets. This survey was created using Google Forms and distributed online on the Portuguese pet information website ‘O Meu Animal’ (https://omeuanimal.com and on social media (i.e., relevant Facebook groups) from December 2018 to January 2019. Participants, consisting of Portuguese residents above 18 years old, agreed with data collection for scientific purposes, and data protection was achieved by excluding personal information (owner’s name) from the analysis after removing repeated entries. The survey’s questions, comprising multiple-choice and checkboxes, included demographic data (region, age, sex, pet types, and number of pets); diet choices and motivations (diet type, justification for the choice, information origin, changes of diet, and optimal body score); feeding practices (snacks, scraps, feeding habits, use of toys, use of puzzles, pet body score, and daily exercise); and finally opinion on 14 sentences on diet, nutrition, and body score to be classified from 1 to 5 as completely agree to completely disagree. Short answers were used to collect numeric information, such as age or number of pets and for an optional suggestion box. North, Center, Metropolitan Region of Lisbon (hereby Lisbon), Alentejo, Algarve, Autonomous Region of Azores (hereby Azores), and Autonomous Region of Madeira (hereby Madeira) were defined according to the Portuguese NUTS II regions. Questions were developed considering a similar survey conducted on pet nutrition and diets [5,6].

On diet types, the following options were included on the survey: (i) commercial pet foods, consisting of regular and widely available formulations, or over-the-counter foods; (ii) therapeutical commercial pet foods, consisting of formulations for specific diseases or medical conditions; (iii) grain free pet foods, consisting of formulations without grains; (iv) natural or organic pet foods, consisting of pet foods using restricted “natural” ingredients and natural preservatives; (v) low carbohydrate pet foods, consisting of formulations, which present a low carbohydrate profile; (vi) vegetarian or vegan pet foods, consisting of formulation using plant-based ingredients; (vii) raw meat diets or pet foods, consisting of uncooked meals mainly comprising animal products; and (viii) homemade diets, consisting of preparing and cooking diets at home. Although all these options were provided, not all were represented in the answers provided by surveyed pet owners.

Statistical analysis was conducted on IBM SPSS Statistics version 24 (IBM, Armonk, New York, USA) through descriptive statistics and Kruskal–Wallis one-way analysis of variance (H), for comparing between categories of numerical variables, and Likelihood Ratio (LR), to compare between categorical variables. H_0_ was that there were no significant differences between the categories being compared by the test, considering an α = 0.05. Statistical analysis on diet type was conducted by grouping diet types into three categories: commercial (commercial diet), alternative (homemade, low carbohydrate, grain free, organic, or natural), and therapeutic (therapeutic commercial diet). Invalid responses were excluded from the analysis.

## 3. Results

### 3.1. Characteristics of the Participants and Companion Animals

A total of 74 valid responses were obtained. Most of these respondents were from the North (55.4%), corresponding to an overrepresentation of this region compared to the Portuguese population (Table 1). The respondents were mainly female (90.5%) with a mean age of 33 years old (ranging from 19 to 65 years old), similar to previous surveys [3]. Respondents reported mainly having dogs (70.3%) and cats (63.5%), with 12.2% having other species (e.g., fish, reptiles, and small mammals). Regarding the cohabitation of multiple species in a single household: (i) 27.0% of households reported having both cats and dogs; (ii) 6.8% reported having cats, dogs, and other species; (iii) 2.7% reported having cats and other species; and (iv) 2.7% reported having dogs and other species. The median number of cats per participant was 1 (0–5) and of dogs 1 (0–7).

### 3.2. Diet Choices of Portuguese Owners

**Figure 1:**
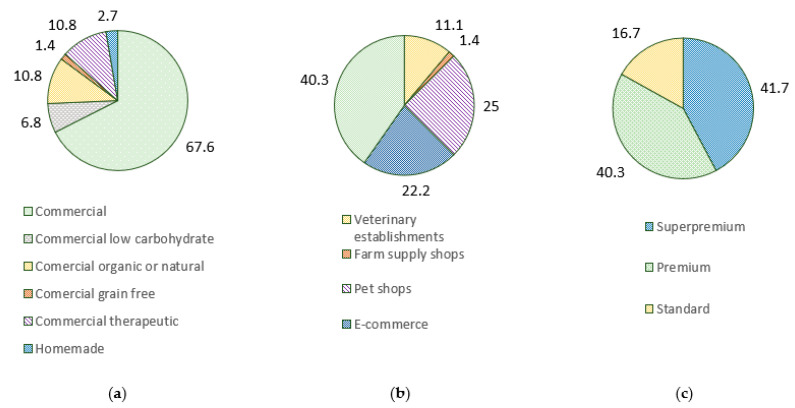
Percentage (%) of reported diet choices regarding type of diet (**a**), place of purchase (**b**), and diet quality (**c**) used by Portuguese owners responding the survey.

Respondents reported primarily feeding commercial diets (67.6%), followed by alternative commercial diets (19.0%), mainly comprising diets labeled as organic or natural (10.8%) (Figure 1). The self-reported diet quality was super-premium (41.7%) and premium (40.3%). Most diets were purchased in supermarkets (40.3%), followed by pet shops (25%). Choice of diet was related neither to region (LR_10_ = 7.912, *p* = 0.637) nor purchasing establishment (LR_10_ = 16.826, *p* = 0.078) but was related to self-reported quality (LR_6_ = 17.105, *p* = 0.09), namely higher self-reporting of super-premium brands in alternative diets (75.0%). The median monthly cost of feeding companion animals was EUR 30 (EUR 3–150), depending on diet choices (H_2_ = 9.190, *p* = 0.010), with significantly higher costs of therapeutical diets (EUR 60) compared to commercial diets (EUR 30, *p* = 0.020). No effect on monthly costs was observed for self-reported diet quality (H_3_ = 1.368, *p* = 0.713) or place of purchase (H_5_ = 7.820, *p* = 0.166).

Respondents were free to comment on their choice of pet foods. Comments related to the use of commercial diets included “low prices”, “recommended by the veterinarian”, “the pet likes it”, “good quality”, “easier to acquire in my area of residence”, and “recommend by the national consumer association”. Comments on alternative diets included “better for digestion”, “recommended by the veterinarian”, “I try to feed the healthiest pet food”, and “general knowledge”. Therapeutical diets were mostly related to veterinarian recommendations and in the management of health conditions, such as bladder stones and feline cystitis. Overall, commercial diets seem to be mostly related to practical concerns (e.g., availability, price, and palatability), while alternative diets are motivated by greater health concerns (e.g., better digestion and healthier food). No information was collected on brands of pet food used.

### 3.3. Feeding Habits of Portuguese Owners

**Figure 2:**
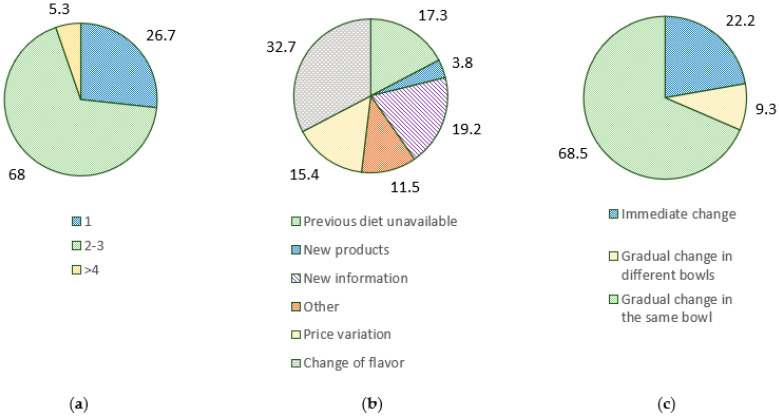
Feeding habits of Portuguese owners responding the survey regarding the number of daily feedings (**a**), reasons behind diet changes (**b**), and the way that diet change is conducted (**c**).

Most respondents reported feeding two to three times per day (68.0%) following the instructions on the label to dose the food (60.5%), supplying also snacks or treats (77.3%) and table scraps (38.7%) (Figure 2). Toys releasing food (60.8%), and puzzles or labyrinths (87.8%) were not used by most pet owners. Most respondents did not change the diet in a period equal or less than three months (68.1%), and this was independent of diet type preferences (LR_4_ = 4.734, *p* = 0.316). Diet changes were mainly motivated by the intention of changing the flavor (32.7%) or by the owner obtaining new information (19.2%). Changes were mainly conducted by gradual mixing of the old and new diet in the same bowl (68.5%). There were significant differences in the use of toys releasing foods (LR_2_ = 9.868, *p* = 0.007), puzzles or labyrinths (LR_2_ = 7.030, *p* = 0.030), and feeding of snacks and treats (LR_2_ = 7.489, *p* = 0.024) and diet type. These results are highly influenced by sample size (*n* = 74) and, mostly, the lack of use when feeding a therapeutical commercial diet, despite suggesting a small trend for higher use when feeding alternative diets (Table A5, Appendix B).

### 3.4. Exercise, Affective Feeding, and Body Condition Score

Most pet owners spent 30 min to one hour playing or walking their pets (29.3%), with similar results for 15 to 30 min (25.3%) and 1 to 2 h (25.3%), with 10.7% of owners spending more than 2 h a day with their pets. Respondents agree that feeding strengthens their affective relationship (96.0%). When shown an image of body condition score (1-very thin to 5-extremely obese), pet owners identified optimal body condition with a median score of 3 (1–4) and self-classified their animals as 3 (2–4). No significant differences were found between body condition score and how the pet owners determined the amount of food (H_2_ = 0.805, *p* = 0.669), the number of feedings per day (H_3_ = 5.583, *p* = 0.134), the time spend playing with the animal (H_4_ = 1.347, *p* = 0.853), feeding of snacks (H_1_ = 0.620, *p* = 0.431), or feeding of table scraps (H_1_ = 0.348, *p* = 0.555). No significant difference was also found between body conditions scores for diet types (H_2_ = 0.915, *p* = 0.633).

### 3.5. Reasons for Pet Food Choices and Sources of Information on Healthy Pet Diet

**Figure 3:**
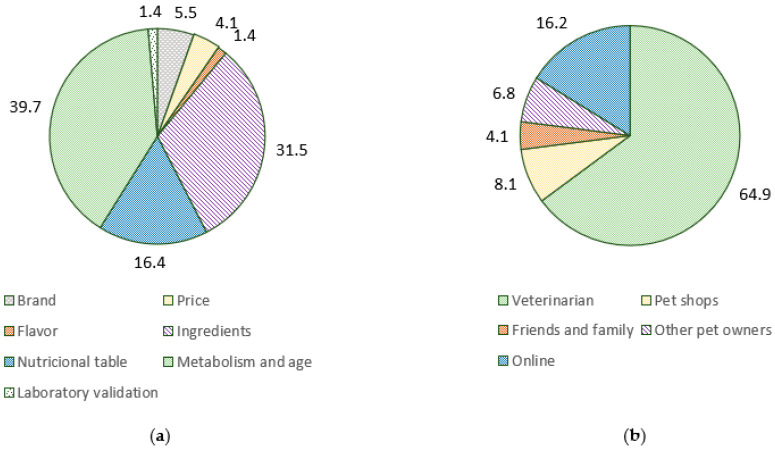
Major factors involved in the choice of pet food (**a**) and sources of information for this choice (**b**).

For pet owners, the most important factors in the choice of pet diets were the adequacy for metabolism and age of their pet (39.7%), followed by the ingredients list (31.5%) (Figure 3). Most respondents report having talked about the pet’s diet with their veterinarian (82.7%), and the majority intend to do so again in the future (84.0%). Indeed, most respondents based their pet food choices on information obtained from the veterinarian (64.9%), followed by information online (16.2%). Diet type was not influenced by the most important factors considered in the choice of pet diets (LR_14_ = 15.039, *p* = 0.375) nor the source of the information on pet nutrition (LR_8_ = 10.458, *p* = 0.234). Despite the lack of significance, pet food choice based on ingredient list seems to influence diet choice, with pet owners choosing less frequently regular commercial diets (47.8%) and choosing more often organic or natural (26.1%), low carbohydrates (8.7%), and homemade (4.3%) diets. Conversely, pet owners obtaining information from online sources preferred commercial diets (83.3%) (Table A3, Appendix B). Having previously consulted the veterinarian about pet diet was related to diet choice (LR_2_ = 6.880, *p* = 0.032), corresponding to 78.0% of pet owners using commercial diets, 87.5% using therapeutical diets, and 100% using alternative diets.

### 3.6. Pet Owners’ Opinions on Pet Food

**Figure 4:**
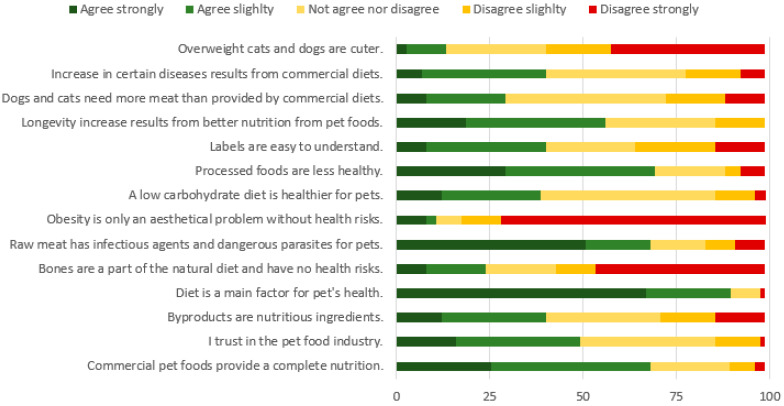
Opinion of Portuguese pet owners on statement regarding pet foods.

Regarding commercial diets, most owners agree that pet foods provide complete nutrition (68.0%), that they trust the pet food industry (49.3%), and that increased pet longevity is a result of proper nutrition provided by pet foods (56.1%) (Figure 4). However, an important percentage (40.0%) believes that commercial diets are related to the increase in certain diseases, such as cancer or chronic diseases. Indeed, 89.4% of pet owners agree that diet is a major factor in their pet’s health. While the majority of owners understand the labels in pet food easily (40.0%), a considerate amount still finds it somewhat difficult (34.6%). Most respondents disagree that they find overweight pets cuter (58.6%) and that pet obesity is a purely aesthetical problem (81.4%), which reveals a high sensibility to this topic. Regarding alternative diets, most pet owners agree that by-products are nutritious (40.0%), that raw meat contains dangerous pathogens and parasites (68.0%), and that ingestion of animal bones has health risks (56.0%). Opinions are divided regarding the need for more meat in commercial diets, with 29.3% of owners agreeing with this need, 26.7% disagreeing, and most not having an opinion (42.7%). Regarding low carbohydrate diets, many agree that this is a healthier choice (38.7%), with most do not having a strong opinion on the topic (46.7%). Despite the support for commercial pet foods, as previously mentioned, pet owners strongly believe that processed foods are less healthy (59.3%). All tests between diet types are non-significant apart from “Overweight cats and dogs are cuter” (H_2_: 6.328; *p* = 0.042), for which owners feeding therapeutically diets strongly disagree when compared to other diet types. Averages and medians for each question are presented in Table A4, Appendix B.

### 3.7. Information Needs of Pet Owners Regarding Pet Diets

**Figure 5:**
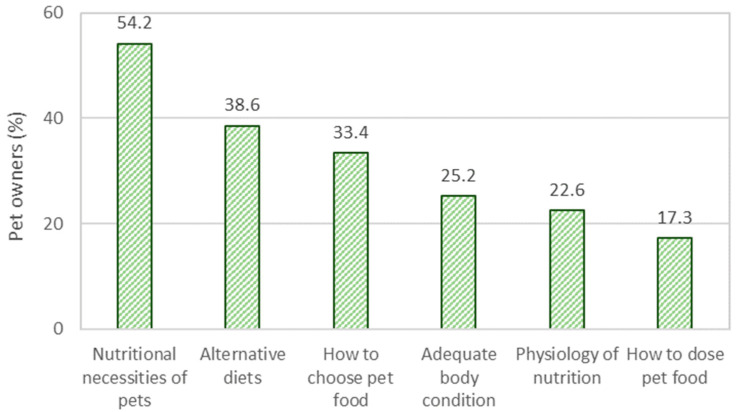
Self-reported information needs on pet feed and nutrition of Portuguese pet owners.

Pet owners were asked to select the topics on pet nutrition and feeding that they felt more interested in learning (Figure 5). Most pet owners wanted to learn more about the nutritional necessities of pets (54.2%), followed immediately by alternative diets (38.6%) and how to choose pet food (33.4%).

## 4. Discussion

### 4.1. Demographics and Pet Food Types

A total of 74 valid responses were obtained in the current survey on pet nutrition and feeding habits conducted in Portugal. Most respondents identified as female, possibly related to the higher tendency of females to respond to surveys [16], while the median age of 33 years old was below the national median age of 45 years old [15], likely due to the online dissemination medium mostly being used by a younger generation [17]. Dogs (70.3%) were more frequent pets than cats (63.5%), in agreement with a previous report of Portuguese veterinary practices in 2016, where dogs comprised 58.6% of business followed by cats (38.6%) [18]. Most households only owned one cat or one dog. Moreover, 27.0% of families owned both cats and dogs, a lower number than the previously reported in Portugal (36.9%) [3], likely due to a different regional distribution of responses.

Most pets were fed commercial diets (67.6%) followed by alternative diets (19.0%), therapeutic diets (10.8%), and homemade diets (2.7%). However, only 10.1% of owners reported using alternative or homemade diets in a study conducted in Portugal in the previous year [3], likely due to different regional distribution and sample sizes. Similarly, only 12.8% of pets in the USA are fed alternative diets and 3.8% homemade diets [5]. Supermarkets are the preferred place of purchase for Portuguese pet owners, with a median monthly spending of EUR 30, contrary to the preference for specialty stores in the USA [5]. While only 41.7% of pet owners self-report quality of diet as super premium, the self-report quality classifications have no influence on monthly costs with animal feeding (H_3_ = 1.368, *p* = 0.713). Moreover, 75.0% of pet owners feeding alternative diets consider these diets as super-premium despite these diets not having a superior cost, which likely translated into a perceived higher quality of alternative diets by their advocates. Indeed, Italian owners classified pet foods as higher quality based on the perceived presence of “natural ingredients” [4]. On the other hand, pet owners using therapeutic diets report having twice the monthly cost of other diets, which is EUR 60.

### 4.2. Feeding Habits, Exercise, and Body Condition Score

Regarding the feeding habits, most participants follow the instructions on the label on the amount of food (60.0%), providing two to three feedings per day (68.0%), and feeding snacks or treats (77.3%) and table scraps (38.7%). In Massachusetts, USA, most owners also feed their pets twice a day (52.0%) and feed treats daily (62.0%), with 30% following label directions regarding the amount of food [19]. Feeding of snacks or treats and table scraps is correlated with excess weight in dogs [20,21,22] and thus should not exceed 10% of the daily calory intake, as recommended by The World Small Animal Veterinary Association (WSAVA) [23]. Similarly, feeding more than twice a day is related to dog obesity [21,22]. Only 44 to 49% of owners correctly prepare the proper amount of pet food when following the label’s instructions [24].

Pet owners correctly identified the ideal BCS and self-reported their pet’s BCS as 3/5 (ideal) and recognized the dangers of obesity to health. However, owners are known to inaccurately report BCS for their own pets, even when following a chart and recognizing the health risks of obesity, likely due to their own ‘normalization’ of the body shape as well as reluctance to accept that their pet has excess weight due to its negative associations [25]. Moreover, 65.3% of pet owners spend over 30 min daily interacting with their pet, which is known to reduce the risk of obesity [21,22]. The use of food puzzles could also have positive effects on animal health and behavior, such as promoting exercise and reducing anxiety [26,27]. However, most respondents do not use toys releasing food (60.8%), and puzzles or labyrinths (87.8%) as environmental enrichment. This is similar to their use by California cat owners (30%) [14] and higher than previous studies on cats conducted in Lisbon, Portugal (4.6%) [28].

### 4.3. Factors Influencing Pet Food Purchasing Decision

The most important factors on the choice of pet diet were the adequacy for metabolism and age (39.7%), followed by ingredient list (31.5%). Likewise, health and ingredient list were the major factors identified in the USA [19]. Ingredients used in pet food are safe and are used to produce an appropriate balance between nutrients, which truly represents the quality of the feed [29]. Evaluation of the ingredient list is meaningless when neglecting their proportions, specific nutrient profiles, and digestibility. Conversely, consumers interpret pet food ingredient lists based on an expectation of what should constitute an optimal diet (e.g., the ancestral species’ diet and human grade) and what is perceived as a low-quality ingredient (e.g., grains and by-products) [10]. The presence of “natural ingredients” is perceived as higher quality [4] and can justify the observed shift to alternative pet diets. Indeed, valorization of the ingredient list seems related to a preference for alternative or homemade diets, although not significant due to the limited number of observations. A complete and balanced diet for the proper metabolism and age as well as reference to validated animal feeding tests should instead be used to attest the suitableness of the diet [30].

Despite the ingredient list being the second most important factor in the choice of pet foods, most owners have already discussed pet diets with their veterinarian (82.7%) and intend do so in the future (84.0%). Veterinarians are still the major source of information regarding pet diets (64.9%), followed by online information (16.2%), similar to the USA, where information was mainly obtained from veterinarians (43.6%) and the internet (24.6%) [5]. Curiously, significantly more respondents feeding therapeutic and alternative diets reported having consulted with their veterinarians compared to owners feeding commercial diets, which could translate to a greater interest in providing healthier diets. Conversely, the internet is the second most important source of information (16.2%) and is related to the consumption of commercial diets (83.3%), which could be related to online advertisement. Most owners do not change pet diets in periods ≤3 months (68.1%), with this switch mostly motivated by the intention to change the food flavor (32.7%) followed by choosing a diet based on newly acquired information (19.2%), and mostly conducting by mixing both diets in the same bowl (68.5%). A previous study has identified that dog owners worry about their pet’s eating experience, including the taste and variety of diets [31]. Additionally, owners may not believe that a single nutritionally complete product could satisfy the nutritional necessities of their pets in the long-term, urging them to switch pet foods [32]. Therefore, the contents of the newly acquired information, either from the veterinarian or the internet, can have an important impact on the diet of companion animals, which requires further attention.

### 4.4. Perceptions and Information Needs

There are conflicting beliefs regarding commercial foods, with respondents agreeing that pet foods provide complete nutrition (68.0%), increased longevity (56.0%), and can be trusted (49.3%), but also that pet food increases the risk of certain diseases (e.g., cancer) (40.0%), that more meat is required (29.3%), and that low carbohydrate diets are healthier (38.7%). These perceptions are well-related to the high importance given to the ingredient list (31.5%) when choosing a pet food, second only to adequacy for metabolism and age. Similarly, a study conducted in Italy identified the presence of “natural” ingredient claims in pet food being the most important quality indicator used by pet owners [4]. A survey on California found an association between beliefs about pet food and diet type, with pet owner using alternative diets presenting more concerns about commercial pet foods [6]. As previously mentioned, alternative diets (including commercial choices) are considered higher quality (i.e., super-premium) despite being independent of price. While only 19.0% of respondents feed alternative diets, 38.6% show interest in learning more about this diet type, second only to learning more about nutritional necessities of pets (54.2%). This interest may originate in the belief that these diets are healthier, contain higher quality ingredients, or comply with the ancestral diet of precursor species [33]. Therefore, a growth of alternative diets in the Portuguese public is expected according to the opinions of respondents.

## 5. Conclusions and Recommendations

The current study revealed that most Portuguese respondents feed their companion animals commercial diets (67.6%) bought in supermarkets (40.3%). Despite limitations regarding the number of respondents (*n* = 74), overrepresentation of some regions, and predominance of female respondents, the study reveals a growing trend in the consumption (19.0%) and interest (38.6%) for alternative diets (e.g., natural diets and organic diets). Respondents identify alternative diets as super-premium, despite not reporting an increase in monthly expenses (EUR 30), possibly because the ingredient list is considered the second most important factor when choosing pet foods (31.5%). The importance of the ingredient list is also translated into the beliefs of many respondents that pet food should consist of a higher proportion of more meat (29.3%) and less carbohydrates (38.7%). Information about pet foods mostly originates from the animal’s veterinarian (64.9%), followed by the internet (16.2%). Surprisingly, more respondents feeding alternative diets have discussed pet diet with veterinarians, while commercial diets are related to higher preponderance of online content. The main motivation of pet owners feeding alternative diets was to provide a perceived healthier diet based on expectations over the ingredients used, being evaluated by seeking veterinarian advice. As in Portugal, growing trends of alternative diets have been reported for other countries (e.g., USA and Italy) where surveys have been conducted.

The growing interest in alternative diets and the importance of the ingredient list on pet food choices will likely result in dietary changes over the next years unless conventional brands adapt their marketing strategies. While alternative diets can be complete and balanced, quality can vary and novel ingredients or combinations may lead to nutritional deficiencies (e.g., cardiomyopathy). More research is needed on unconventional nutrient sources and reputable feeding tests of alternative diets. Although pet’s diets, especially of dogs, were historically comprised of prey animals and then of food scraps or homemade foods, there is no evidence that these diets provided adequate nutrition [32]. Commercial pet foods were intended to provide complete nutrition at a low cost and high convenience, which, along with improvements in husbandry and healthcare, have resulted in a greater living expectancy in pets. While generally considered safe, pet food recalls due to presence of contaminants [34] and the use of some pet food ingredients and/or additives, such as the recently ban on ethoxyquin in the European Union [35], have made consumers less trustful of common over-the-counter pet food formulations. Moreover, companion animals are now considered as family members [36], increasing pet owners’ interest in their wellbeing and in seeking healthier diets [3]. Despite the good intentions, the use of alternative diets have often been accompanied by nutritional imbalances, such as deficiency in calcium in raw diets for dogs [37] or deficiency in taurine in vegan diets for cats [7]. In alternative commercial pet foods, nutritional imbalances represent failures in formulation due to knowledge gaps or inadequate industrial processes, as animals have no ingredient requirements but nutrient requirements, which can be met by an infinite number of ingredient combinations. Indeed, a fast shift to alternative diets may be disruptive to the pet food industry, having to adapt processes and equipment, depending on the availability and price of new feedstocks, and increasing the environmental footprint by competing with ingredients in high demand for human consumption. One example of such a failure was seen with the increased incidence in dilated cardiomyopathy in susceptible dogs eating grain free diets, thought to have originated in a taurine deficiency as a result of the use of new carbohydrate sources, which may have reduced the amount, reduced the bioavailability; increased the excretion; or altered the metabolism of taurine or of its precursors, methionine and cysteine, in the diet [9]. Greater interest in alternative diets and new nutrient sources will likely motivate research and avoid such outcomes in the future.

Due to the initial variation in quality, veterinarians should strengthen their oversight over pet diets, for instance, by following Parr and Remilland’s recommendations on how to evaluate alternative diets [32]. Since many pet owners will seek online information on pet foods, it is recommended that veterinarians also provide recommendations on content quality and discuss the findings. Health is not the only concern of pet owners when choosing pet foods, who also weight the eating experience (e.g., flavor). Therefore, veterinarians could advise on the use of toys, puzzles, and labyrinths to provide environmental enrichment during feedings and to diversify the pet’s eating experience without changing or adding elements to the diet. Despite personal opinions and beliefs, veterinarians should be receptive to discussing and accommodating pet owners’ choices, ensuring adequate nutrition and health promotion.

## Figures and Tables

**Table 1:** Population percentage in survey responses (%) compared to real population distribution (%), adapted from Pordata (2018) [15].

Region	*n*	Responses (%)	Population (%)
North	41	55.4	34.7
Center	16	21.6	21.6
Lisbon	7	9.5	27.6
Alentejo	2	2.7	6.9
Algarve	0	0	4.3
Azores	2	2.7	2.4
Madeira	6	8.1	2.5

## Data Availability

Data available on request due to privacy restrictions.

## Appendix A

**Table A1:** Survey questions.

Category	Question	Response
Demographic data	I agree with the participation in the study and anonymous collection of data (Consent form).	Yes/No
	Name	Open question
	Sex	Male/Female
	Region	North, Center, Lisbon, Alentejo, Algarve, Madeira, Açores
	Age	Open question
	What companion animals do you have?	Dog, Cat, Others
	How many dogs do you own?	Open question
	How many cats do you own?	Open question
Pet food choices and habits	What type of diet do you feed your pet?	Commercial, Commercial low carbohydrate, Commercial organic or natural, Commercial grain free, Commercial therapeutical, Homemade
	Why do you choose to use this type of pet food?	Open question
	Where do you obtain information to choose the companion animal’s diet?	Veterinarian, Pet shops, Friends and family, Other pet owners (in person), Online (websites, social media, forums)
	Have you talked about your pet’s diet with your veterinarian?	Yes/No
	Do you intend to talk about your pet’s diet with your veterinarian in the future?	Yes/No
	What do you think is most important when choosing the diet of your companion animal?	Brand, Price, Flavor, Ingredients, Nutritional table, Metabolism and age, Laboratory validation, I do not buy
	Where do you buy pet food?	Veterinarian, Farm supply stores, Pet shops, E-commerce, Supermarkets, I do not buy
	What type of pet food do you usually buy?	Standard (low range), Premium (middle range), Super-premium (high range), I do not buy
	Do you usually switch pet foods (at each 3 months or less)?	Yes, No, I do not buy
	What is the factor leading you to switching pet foods?	Previous diet unavailable, New products, New information, Price variations, Change of flavor, Others, I do not buy
	How do you usually conduct a change of pet foods?	Immediate change, Gradual change in a different bowl, Gradual change in the same bowl, I do not buy
	In average, how much do you spend each month feeding your pet (€)?	Open question
	Based on the picture presented above, what do you think is the ideal body condition score for a cat or dog?	1, 2, 3, 4, 5
	Do you feel that feeding is important to reinforce the affective connection with your pet?	Yes/No
Pet feeding habits	Do you usually feed treats or snacks for cats or dogs?	Yes/No
	Do you usually feed foods for human consumption (e.g., from your plate, food scraps, etc)?	Yes/No
	How do you feed your pet?	Fill the bowl, Dosing according to the label, I do not use
	How many times per day do you feed your pet?	1, 2–3, 4–5, >5
	Do you use toys which release foods?	Yes/No
	Do you use food bowls with puzzles or labyrinths which reduce the food ingestion velocity?	Yes/No
	Based on the picture above, what is the body condition score of you dog or cat?	1, 2, 3, 4, 5
	How long do you spend each day playing or walking your pet?	<15 min, 15–30 min, 30–60 min, 1–2 h, >2 h
Pet owner’s opinions	Commercial pet foods provide a complete nutrition.	Classify the sentences according to your degree of agreement: Strongly agree Agree slightly Do not agree or disagree Disagree slightly Strongly disagree
	I trust in the pet food industry.	
	Byproducts are nutritious ingredients.	
	Diet is a main factor for pet’s health.	
	Bones are a part of the natural diet and have no health risks.	
	Raw meat has infectious agents and dangerous parasites for pets.	
	Obesity is only an aesthetical problem without health risks.	
	A low carbohydrate diet is healthier for pets.	
	Processed foods are less healthy.	
	Labels are easy to understand.	
	Longevity increase results from better nutrition from pet foods.	
	Dogs and cats need more meat than provided by commercial diets	
	Increase in certain diseases results from commercial diets.	
	Overweight cats and dogs are cuter.	
	What topics related to pet foods and feeding would you like to know more about?	Nutritional necessities of pets, Alternative diets, How to choose pet foods, Adequate body condition, Physiology of nutrition, How to dose pet food

**Table A2:** Online informed consent form.

Consent Form	Responses
You are invited to participate in a research study about pet food diets and practices in Portuguese pet owners. Participants should be over 18 years old. Participation in this study is voluntary and respondents can leave the page at any moment without personal prejudice. The survey takes 25 min to complete and is mainly comprised of multiple-choice and checkboxes, while participants may optionally also write open responses. While the study may not benefit you directly, it will provide essential information on the husbandry and feeding practices in Portugal, which provides essential information to improve pet care at national level. All collected data is confidential and anonymous. Data collected will not be used for commercial purposes, marketing campaigns, or provided to third parties. Data collected in the survey will be solely used for statistical and scientific purposes. Data will be stored safely and only accessed by the investigator for the purposes stated above. By accepting the consent form, I agree with the use of data for non-commercial use.	I agree with the participation in the survey and anonymous collection of data.
	I do not agree.

## Appendix B

**Table A3:** Distribution of pet food diets for groups where the main factor in diet choice is the ingredient list or not, and the main source of information in the choice of pet foods is online content or not.

	Main Factor in Diet Choice		Main Source of Information	
	Ingredients	Not Ingredients	Online	Not Online
Commercial diet	47.8	76.5	83.3	64.5
Home made	4.3	2.0	0.0	3.2
Commercial low carbohydrates	8.7	5.9	8.3	6.5
Commercial organic or natural	26.1	3.9	8.3	11.3
Commercial grain free	0.0	2.0	0.0	1.6
Commercial therapeutical	13.0	9.8	0.0	12.9
N	23	51	12	62

**Table A4:** Scores for pet owners about statements regarding attitudes towards feeding practices of their pets, in a scale varying from 1, strongly agree, to 5, strongly disagree.

	Pet Food Type							
	Total		Commercial		Alternative		Therapeutical	
	Mean ± SD	Median	Mean ± SD	Median	Mean ± SD	Median	Mean ± SD	Median
Commercial pet foods provide a complete nutrition.	2.2 ± 1.0	2.0	2.1 ± 0.9	2.0	2.4 ± 1.1	2.5	2.4 ± 1.1	2.5
I trust in the pet food industry.	2.5 ± 1.0	2.5	2.4 ± 0.8	2.0	2.6 ± 1.1	3.0	2.5 ± 1.3	2.5
Byproducts are nutritious ingredients.	2.9 ± 1.2	3.0	2.8 ± 1.1	3.0	3.1 ± 1.3	3.0	3.0 ± 1.6	3.0
Diet is a main factor for pet’s health.	1.4 ± 0.8	1.0	1.5 ± 0.7	1.0	1.4 ± 1.0	1.0	1.1 ± 0.4	1.0
Bones are a part of the natural diet and have no health risks.	3.7 ± 1.4	4.0	3.8 ± 1.4	4.0	3.3 ± 1.4	3.0	4.1 ± 1.5	5.0
Raw meat has infectious agents and dangerous parasites for pets.	2.0 ± 1.3	1.0	2.1 ± 1.4	1.0	2.1 ± 1.2	2.0	1.8 ± 1.2	1.0
Obesity is only an aesthetical problem without health risks.	4.4 ± 1.2	5.0	4.3 ± 1.3	5.0	4.6 ± 0.8	5.0	4.0 ± 1.4	4.5
A low carbohydrate diet is healthier for pets.	2.6 ± 0.9	3.0	2.6 ± 0.9	3.0	2.6 ± 1.1	2.5	2.8 ± 0.9	3.0
Processed foods are less healthy.	2.2 ± 1.1	2.0	2.1 ± 1.0	2.0	2.1 ± 1.3	2.0	2.5 ± 1.2	2.5
Labels are easy to understand.	3.0 ± 1.2	3.0	3.0 ± 1.2	3.0	3.1 ± 1.3	3.0	2.5 ± 1.1	3.0
Longevity increase results from better nutrition from pet foods.	2.4 ± 0.9	2.0	2.4 ± 0.9	2.0	2.4 ± 0.9	2.0	2.3 ± 1.2	2.5
Dogs and cats need more meat than provided by commercial diets	3.0 ± 1.0	3.0	3.1 ± 1.1	3.0	2.5 ± 0.8	2.0	3.4 ± 1.3	3.0
Increase in certain diseases results from commercial diets.	2.8 ± 1.0	3.0	2.9 ± 1.0	3.0	2.7 ± 1.1	2.5	2.4 ± 0.7	2.5
Overweight cats and dogs are cuter.	3.9 ± 1.2	4.0	3.7 ± 1.2	4.0	4.0 ± 1.0	4.0	4.8 ± 0.5	5.0

**Table A5:** Diet type and the use of toys releasing food, puzzles and labyrinths for food, and snacks and treats.

		Commercial Diet	Alternative Diet	Therapeutical Diet
Use of toys releasing foods	Yes	20	9	0
	No	30	7	8
Puzzle or labyrinths for food	Yes	4	5	0
	No	46	11	8
Use of snacks and treats	Yes	41	14	3
	No	9	2	5
